# Fabrication of Two Polyester Nanofiber Types Containing the Biobased Monomer Isosorbide: Poly (Ethylene Glycol 1,4-Cyclohexane Dimethylene Isosorbide Terephthalate) and Poly (1,4-Cyclohexane Dimethylene Isosorbide Terephthalate)

**DOI:** 10.3390/nano8020056

**Published:** 2018-01-23

**Authors:** Duy-Nam Phan, Hoik Lee, Dongeun Choi, Chang-Yong Kang, Seung Soon Im, Ick Soo Kim

**Affiliations:** 1 Nano Fusion Technology Research Group, Division of Frontier Fibers, Institute for Fiber Engineering (IFES), Interdisciplinary Cluster for Cutting Edge Research (ICCER), Shinshu University, Ueda, Nagano 386-8567, Japan; duynamphan@gmail.com (D.-N.P.); g0024004@yahoo.co.jp (D.C.); 2 Department of Metallurgical Engineering, Pukyong National University, Busan 608-739, Korea; metkcy@pknu.ac.kr; 3 Department of Organic and Nano Engineering, College of Engineering, Hanyang University, Seoul 133-791, Korea; imss007@hanyang.ac.kr

**Keywords:** isosorbide, poly (ethylene glycol 1,4-cyclohexane dimethylene isosorbide terephthalate), poly (1,4-cyclohexane dimethylene isosorbide terephthalate), nanofiber, polyester

## Abstract

The thermal and mechanical properties of two types of polyester nanofiber, poly (1,4-cyclohexanedimethylene isosorbide terephthalate) (PICT) copolymers and the terpolyester of isosorbide, ethylene glycol, 1,4-cyclohexane dimethanol, and terephthalic acid (PEICT), were investigated. This is the first attempt to fabricate PICT nanofiber via the electrospinning method; comparison with PEICT nanofiber could give greater understanding of eco-friendly nanofibers containing biomass monomers. The nanofibers fabricated from each polymer show similar smooth and thin-and-long morphologies. On the other hand, the polymers exhibited significantly different mechanical and thermal properties; in particular, a higher tensile strength was observed for PICT nanofiber mat than for that of PEICT. We hypothesized that PICT has more *trans*-configuration than PEICT, resulting in enhancement of its tensile strength, and demonstrated this by Fourier transform infrared spectroscopy. In addition, PICT nanofibers showed clear crystallization behavior upon increased temperature, while PEICT nanofibers showed completely amorphous structure. Both nanofibers have better tensile properties and thermal stability than the typical polyester polymer, implying that they can be utilized in various industrial applications.

## 1. Introduction

Biomaterials are a subject of great interest, particularly renewable raw materials, as sustainable development policies tend to expand with decreasing fossil fuel reserves and growing concern for the environment [1,2]. Eco-friendly materials, polymers that replace a part of the petroleum monomer with renewable resources, bring significant contributions to new engineering plastics in light of the wider range of disposal options and reduced environmental impacts they offer [3,4]. 1,4:3,6-dianhydrohexitol is one of the few inexpensive difunctional monomers available from renewable resources derived from biomass such as wheat, sugar, and corn [5]. Isosorbide is a stereoisomer of 1,4:3,6-dianhydrohexitol, and, due to its accessible and affordable polymer synthesis, it is the most favored candidate for renewable monomers used in addressing environmental issues [6]. Its unique molecular structure and chirality enhance the glass transition temperature and transparency of the resulting polymers [7]. Therefore, numerous studies have been devoted to the synthesis and characterization of polyesters derived from isosorbide. Recently, we proposed two kinds of terephthalic acid-based polyesters containing isosorbide monomer: the terephthalic acid-based terpolyester of ethylene glycol (EG), isosorbide (ISB), and 1,4-cyclohexane dimethanol (CHDM) (PEICT) [7], and the terephthalic acid-based copolyester of ISB and CHDM (PICT) [4]. Both polyesters show very high thermal resistances with high mechanical properties due to the rigid molecular structure and chirality of isosorbide compared to aliphatic copolyesters.

Nanofibers (NFs) are characterized by extremely long length, small diameter, large surface area per unit mass, and small pore size providing a high surface-to-volume ratio [8]; they are utilized in various applications such as filtration, tissue engineering scaffolds, membranes, antibacterial materials, and photocatalysts [9,10]. The most commonly utilized method for fabricating NFs is the electrospinning technique; this is one of the most effective, straightforward, and facile methods for producing various polymeric fiber sheets of nanometer to micrometer diameter and with a large surface area to volume ratio [11,12]. A wide variety of polymeric materials have been spun into nanofibers via electrospinning, and their properties and practical applications have been studied [13,14]. However, extensive studies of the nanofiber form of copolyesters containing the biomass monomer isosorbide have not yet been conducted. In our previous report [3], we fabricated PEICT nanofiber and documented its enhanced thermal properties and elongation for the first time; however, the properties of PICT have not yet been investigated.

Herein, we fabricated the novel nanofiber PICT via electrospinning and compared it with electrospun PEICT nanofiber. The significant compositional difference between PICT and PEICT is the presence of ethylene glycol in PEICT, which makes for different mechanical, crystallinity and thermal properties in the nanofiber. We optimized the fabrication conditions of both nanofiber and confirmed their morphologies and diameters by scanning electron microscopy (SEM) and image analysis (ImageJ). The diameters of both PEICT and PICT nanofibers tended to increase as the concentration of the polymer increased. Chemical compositions were confirmed by Fourier transform infrared spectroscopy (FT-IR), and the thermal and mechanical properties were investigated via universal testing machine (UTM) and differential scanning calorimetry (DSC), respectively. The PICT nanofiber had higher mechanical properties in terms of tensile stress and strain, which comes from differences in its structural configuration compared to PEICT. In addition, it is worth noting that PICT nanofibers show a crystallization peak at ~140 °C and a melting peak at ~280 °C in their DSC curves, while PEICT nanofibers did not show any characteristic peaks. These results are in good agreement with X-ray diffraction (XRD) results. PICT nanofiber exhibited clear crystallization behavior after annealing at 180 °C for 3 h, while crystallization of PICT was not observed.

## 2. Materials and Methods

### 2.1. Materials

**Figure 1:**
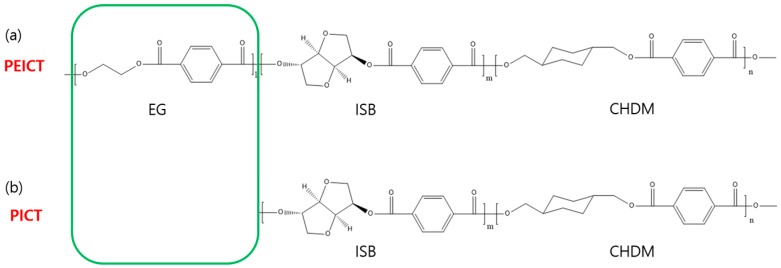
Structures of: (**a**) poly (ethylene glycol 1,4-cyclohexane dimethylene isosorbide terephthalate) (PEICT) and (**b**) poly (1,4-cyclohexane dimethylene isosorbide terephthalate) (PICT). The values of *l*, *m*, and *n* in PEICT were 29.8, 22.7 and 47.2, respectively; and the values of *m* and *n* in PICT were 11.1 and 88.9, respectively. Chemical compositions were determined by NMR in previous reports. Reproduced with permission from [4]. Royal Society of Chemistry, 2015. Reprinted with permission from [7]. American Chemical Society, 2015.

Pellet type PEICT and PICT were supplied by SK Chemicals, Gyeonggi-do, Korea. The PEICT and PICT polyesters were synthesized as reported previously [4,7]. The chemical composition of PEICT was EG, ISB, and CHDM with repeat units in 29.8, 22.7 and 47.2 molar ratio, and that of PICT was ISB and CHDM with repeat units in 11.1 and 88.9 molar ratio, respectively; compositions were confirmed by Nuclear Magnetic Resonance (NMR) in our previous paper [4,7]. The chemical structures of PEICT and PICT are presented in Figure 1. The weight-average molecular weights (*M*_w_) of PEICT and PICT are 51,200 and 46,800 g/mol, respectively, as determined by gel permeation chromatography in our previous paper [4,7]. Trifluoroacetic acid (98%) (TFA) and chloroform (99%) were purchased from Wako Pure Chemical Industries, Ltd. (Osaka, Japan) and were used without any purification.

### 2.2. Characterization

Morphological studies of PEICT and PICT nanofibers were conducted by SEM (JSM-6010LA by JEOL, Tokyo, Japan) with an accelerating voltage of 10 kV. All samples were coated with platinum under a JFC-1200 fin coater (JEOL, Tokyo, Japan) for 60 s before observation. The average diameter and distribution of nanofibers were obtained from SEM micrographs using image analysis software (ImageJ, National Institutes of Health, Bethesda, MD, USA); 50 points were randomly selected within a single SEM image and analyzed to obtain the average value and distribution. Fourier Transform Infrared (FT-IR) spectroscopy on an IR Prestige-21 (Shimadzu, Kyoto, Japan) was employed to determine the types of functional groups present in the nanofibers. Adsorption spectra were recorded at room temperature across the wavelength range of 400 to 4000 cm^−1^. The stress-strain curves of PEICT and PICT mats were obtained using a universal testing instrument (RTC-1250A, A&D CO., Ltd., Tokyo, Japan). Mechanical properties were evaluated in mat type nanofibers spun for one day. All nanofiber mats were prepared using a punch of dog-bone shape, 15 mm (width) × 60 mm (length); the thicknesses of PEICT mats were 0.06 mm (10 wt %), 0.08 mm (14 wt %), and 0.11 mm (18 wt %), while PICT mat thicknesses were 0.05 mm (10 wt %), 0.06 mm (14 wt %), and 0.12 mm (18%). Tensile tests were performed under standard conditions with a 10 N load cell at an extension rate of 10 mm/min and a gauge length of 30 mm. Differential scanning calorimetric (DSC) curves were recorded with a Thermo plus DSC 8230 (Rigaku Co., Ltd., Tokyo, Japan) instrument across the temperature range of 40 to 300 °C with a heating rate of 10 °C/min in N_2_ atmosphere. All PEICT and PICT nanofiber samples were measured three times to ensure the accuracy of results. Wide-angle X-ray diffraction (XRD) patterns were collected at room temperature using a R-AXIPSDS 3C (Rigaku Co., Ltd., Tokyo, Japan) operating at 40 kV, 300 mA using nickel-filtered CuKα radiation; diffraction was detected over an angular range of 5° to 50°. The prepared PEICT and PICT nanofiber sheets were annealed in an air atmosphere at 180 °C for 1 h (heating rate was 1 °C/min) in an electric furnace (NHV-1515D, Motoyama, Co., Miyagi, Japan).

### 2.3. Electrospinning

Different concentrations of PEICT and PICT ranging from 6 to 20 wt % were prepared, each with a 3:1 weight ratio of chloroform/TFA. All solutions were stirred vigorously for 24 h to fully dissolve the samples. In electrospinning, a high voltage is applied to a droplet of the solution that rests on a sharp conducting tip. As a result of molecular ionization or charge redistribution, a Taylor cone is formed and a jet of the solution is extracted. The formed jet is then accelerated by the electric field and collected onto a substrate. An electrospinning apparatus equipped with a high-voltage power supply (Har-100*12, Matsusada Co., Tokyo, Japan), which is capable of generating voltages up to 100 kV, was used as the source of the electric field. The prepared spinning solution was loaded into a 20 mL volume syringe with capillary tip having inner diameter of 0.8 mm and mounted on a programmable syringe pump (KDS-100, KD Scientific, Holliston, MA, USA). The positive terminal of the power supply was connected to the needle, while the negative one was linked to a metallic drum (collector). For all electrospinning procedures, a voltage of 13 kV was applied, the tip-to-collector distance was 18 cm, and the environment was room-temperature with ~40% humidity.

## 3. Results and Discussion

### 3.1. Morphology of PEICT and PICT Nanofibers

**Figure 2:**
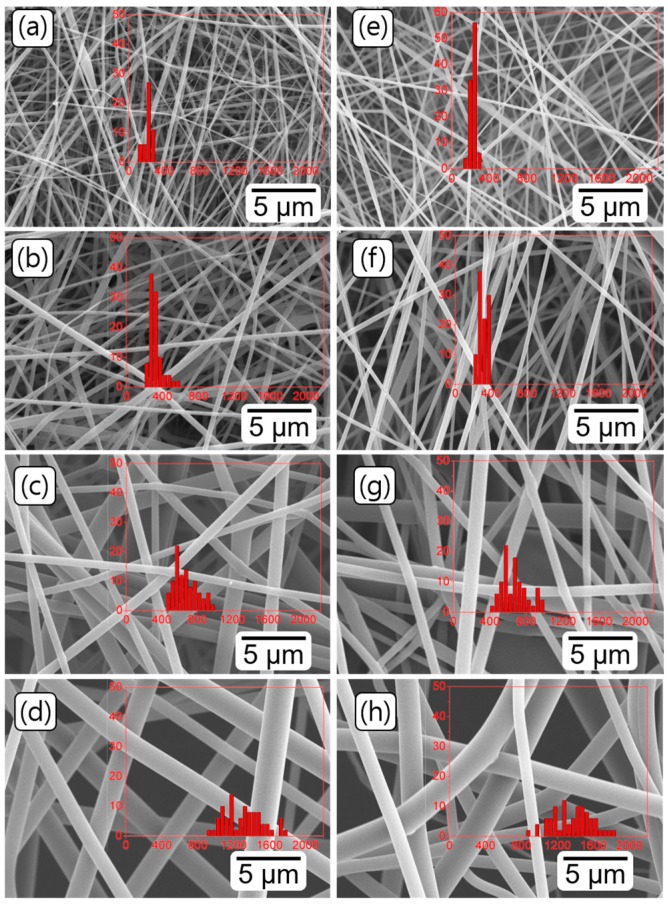
SEM images of the electrsopun nanofibers of PEICT and PICT with different concentrations. The left column shows the morphology of PEICT nanofibers at: (**a**) 6; (**b**) 10; (**c**) 12 and (**d**) 18 wt %. The right column shows PICT nanofibers at: (**e**) 6; (**f**) 10; (**g**) 12 and (**h**) 18 wt %. Insert images present size distribution of nanofibers.

The structure and morphology of the electrospun PEICT and PICT nanofibers were examined by SEM. Both nanofibers showed a bead-free, smooth surface, randomly oriented and uniform structure, as presented in Figure 2. Notably, there was no significant difference in morphology between PEICT and PICT nanofibers. The diameter of nanofibers increased with increasing viscosity, which related to concentration. In a highly viscous solution, densely entangled polymer chains are ejected from the electrospinner tips; consequently, as concentration increased, the nanofibers became thicker and their diameters had wider distributions. In our previous report, the diameter of PEICT nanofibers fitted a power law of the PEICT concentration [3]. The PICT nanofibers exhibited a similar trend (Figure S1). For both polymers, the thinnest fibers were achieved at 6 wt % polymer concentration, resulting in diameters of 219 ± 41 nm for PEICT and 233 ± 31 nm for PICT. The diameter gradually increased and its distribution widened with increasing concentration, finally reaching up to 1695 ± 227 nm for PEICT and 1776 ± 231 nm for PICT nanofiber at 20 wt % polymer.

### 3.2. Chemical Structures and Mechanical Properties of PICT and PEICT Nanofibers

**Figure 3:**
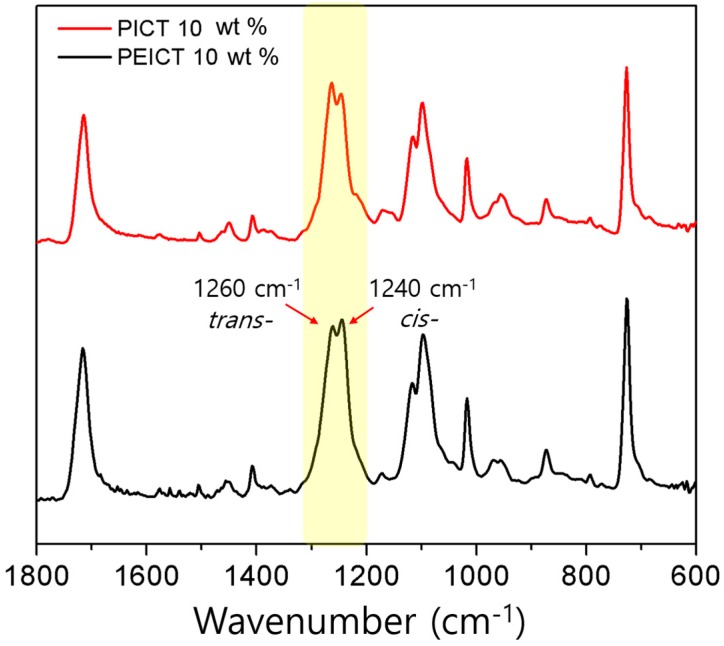
Representative FT-IR spectra for the electrospun PEICT and PICT nanofibers.

One of the most widely utilized polymers is poly (1,4-cyclohexanedimethylene terephthalate) (PCT), an aromatic semicrystalline polyester [15]. The cyclohexylene ring of CHDM flips from e,e-*trans* structure to a,a-*trans* at room temperature via twisted-boat conformation, enhancing chain mobility [16]. This allows for relaxation from its conformational transition, resulting in ductility along with other outstanding properties. Since our two polymers, PEICT and PICT, are based on the PCT structure, they also have the ability to ring flip from the *trans* structure. However, in the case of PEICT, incorporating ISB and EG makes a complex polymer that hinders the formation of the *trans* structure. In addition, the unique V-shape structure of ISB makes for an unfavorable crystallization and ring flip structure. The *trans* structure of PEICT and PICT nanofibers (10 wt %) was confirmed by FT-IR spectroscopy. The spectra did not show any distinguishing differences, except for peaks at 1240 and 1260 cm^−1^, which are assigned to C–O stretching in the *cis* and *trans* structures of CHDM-COO-terephthalate (TER), respectively [3]. As presented in Figure 3, the *trans* peak (1240 cm^−1^) of CHDM-COO-TER showed stronger intensity than that of the *cis* conformation peak in PICT nanofiber spectra. On the other hand, in PEICT nanofiber spectra, the peak at 1260 cm^−1^ was reduced to less than that of PICT, indicating deformation of the *trans* configuration of CHDM due to the complex structure of PEICT. Therefore, the *cis* configuration of CHDM is more prevalent than *trans* in PEICT nanofiber.

**Figure 4:**
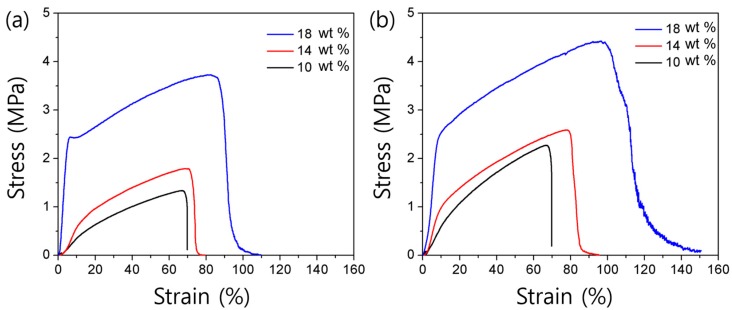
Stress-strain curves measured for: (**a**) PEICT and (**b**) PICT electrospun mats made with polymer concentrations ranging from 10 to 18 wt %.

This configuration difference seems to affect the mechanical strength of the nanofiber. To investigate the differences in mechanical properties between PICT and PEICT, the tensile properties were determined from stress-strain curves of the electrospun nanofibers as the concentration of polymer was varied. A representative stress-strain curve for each nanofiber is presented in Figure 4. The 18 wt % PEICT nanofiber had a tensile strength of 3.73 ± 0.5 MPa and a strain of 82.0 ± 4.8%, while the values for the PICT nanofibers were 4.42 ± 0.6 MPa and 96.3 ± 0.5%, respectively. These correspond to 18% and 17% increases, respectively. At concentrations of 10 and 14 wt %, similar enhancements of tensile properties were also observed (Figure 4). These results indicate that the higher rate of *trans*-configuration in PICT nanofiber makes for more aligned polymer chains than in PEICT nanofiber, consistent with the FT-IR results. In addition, the mechanical properties of both nanofibers were enhanced as solution concentration increased. The stress of PEICT and PICT nanofibers increased from 1.33 ± 0.2 to 3.73 ± 0.5 MPa and 2.27 ± 0.3 to 4.42 ± 0.6 MPa, respectively, as the concentration increased from 10 to 18 wt %.

Generally, nanofibers become thicker as the concentration increases, which is related with solution viscosity [3]. When the spinning time is held equal, a nanofiber mat made with higher concentration is thicker because the solution fabricates thicker nanofibers, as confirmed in Figure 2. For investigation of mechanical properties, all nanofiber mats were prepared with the same spinning times, resulting in relatively thicker mats for higher concentration samples. In thicker mats, each nanofiber can be entangled easily, and the contact area between nanofibers is greater, resulting in increased frictional force. In addition, the polymer chains of thicker nanofibers can be easily entangled, which makes for a higher tensile strength of the mat.

### 3.3. Thermal Crystallization Behavior

**Figure 5:**
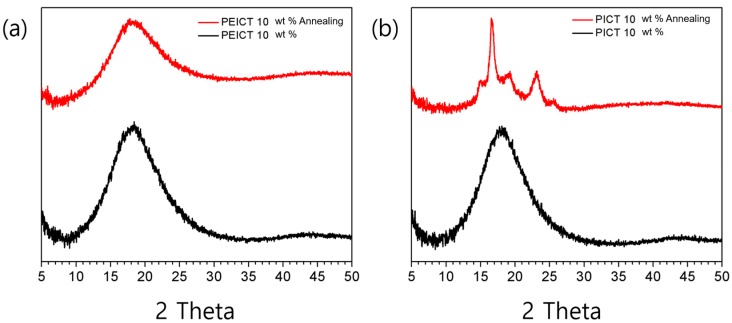
Thermal crystallization behavior before and after annealing at 180 °C for 3 h of: (**a**) PEICT; and (**b**) PICT nanofibers.

We also conducted X-ray diffraction studies to compare the crystallization behaviors of each nanofiber. Figure 5 shows the wide-angle X-ray diffraction spectra of PEICT and PICT nanofibers. Both have relatively low crystallinity due to the rapid fabrication process and complicated chemical structures of the polymers. In particular, ISB is unfavorable for crystallization due to its unique V-shape structure [3]. For both PEICT and PICT nanofibers, only a broad peak was observed at 2*θ* = 18°, typically indicating semi-crystalline regions attributable to the ordering of the polymer chains. It is worth mentioning that noticeable peaks appear for PICT nanofiber after annealing at 180 °C for 3 h, while there is no transition to crystalline in PEICT nanofiber due to its complex polymer structure [7].

**Figure 6:**
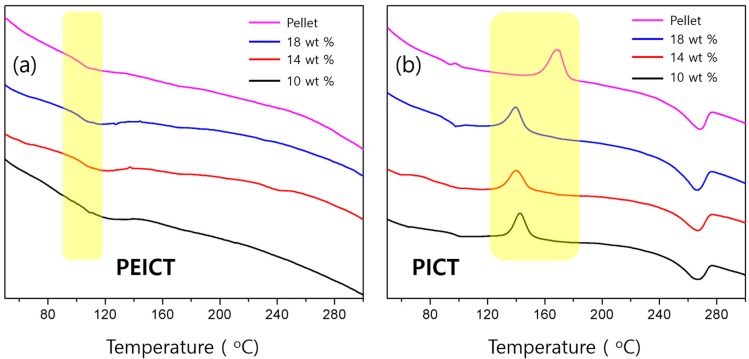
DSC curves of: (**a**) PEICT and (**b**) PICT nanofibers with different polymer concentrations.

The crystallized PICT nanofibers exhibited new peaks at 15.1°, 16.6°, 19,1°, 23.0°, and 25.6°, exactly the same peaks as observed in our previous report [4]. Those peaks correspond to Miller indexes of (011), (010), (110), (100), and (111), respectively [17], indicating clear crystallization behavior for the PICT nanofiber. Interestingly, the fibrous structure was preserved upon annealing in both polymers, as shown in Figure S2, indicating that crystallization of PICT nanofiber does not affect the fiber structure; the fibers can endure the high temperature, indicating high thermal stability. The thermal stability is also discussed in the DSC results. It is known that incorporation of ISB enhances the glass transition temperature because of its rigid molecular structure and chirality [7,18]. In addition, we previously determined that ISB content is directly proportional to the glass transition temperature (*T*_g_) of the polymer [7]. The two types of polyester studied here, PEICT and PICT, showed much higher *T*_g_ values, at ~100 and 90 °C, respectively, than a typical aliphatic polyester, which has a *T*_g_ of around 60 °C (Figure 6). PICT clearly exhibited a crystallization peak and a melting peak, while PEICT samples presented only glass transition behavior, showing that PEICT is a completely amorphous polymer. This supports the crystallization behavior of both polymers as observed by XRD. In a PICT sample, we also observed that thicker nanofibers tended to crystallize more easily. The *M*_c_ peak shifted slightly from 139 to 142 °C as the diameter decreased from ~1700 to ~300 nm (Figure 6b). This phenomenon can possibly be explained by the thicker nanofiber having more free volume to move in, so it more easily forms a crystalline region than does a thinner nanofiber. In contrast, the melting peak arises in the same position at ~270 °C regardless of thickness and form, indicating that melting is entirely determined by polymer.

## 4. Conclusions

The use of polyesters containing biomass monomers in industrial applications has attracted great interest in recent years due to environmental benefits such as energy recovery, CO_2_ reduction, and biodegradability. Herein, we report the successful fabrication of two types of novel polyester nanofiber containing isosorbide, PEICT and PICT, and investigate their mechanical and thermal properties. PICT nanofiber showed higher tensile strength than PEICT nanofiber due to *trans*-configuration in PICT; configuration differences were confirmed by FT-IR. Moreover, the crystallization behaviors of PICT and PEICT also differed, as determined through XRD and DSC. PICT nanofiber crystallized after annealing at 180 °C for 3 h, while PEICT showed only a glass transition temperature at ~100 °C. Both nanofibers demonstrated higher thermal stability than typical polyester polymers; this result is meaningful in aspect of suggesting new biomass polymeric nanofibers and it can be impactful for industrial applications such as tissue engineering, separators, filtration, and scaffolding.

## Supplementary Materials

The following are available online at http://www.mdpi.com/2079-4991/8/2/56/s1.
