# Supplementary material for: Fabrication of Two Polyester Nanofiber Types Containing the Biobased Monomer Isosorbide: Poly (Ethylene Glycol 1,4-Cyclohexane Dimethylene Isosorbide Terephthalate) and Poly (1,4-Cyclohexane Dimethylene Isosorbide Terephthalate)
Source: Nanomaterials (Basel). 2018 Jan 23;8(2):56. doi: 10.3390/nano8020056 (PMC5853689; doi:10.3390/nano8020056)
Supplement: Supplementary file 1 [file nanomaterials-08-00056-s001.pdf]

Article

Fabrication of Two Polyester Nanofiber Types Containing the Biobased Monomer Isosorbide: Poly (Ethylene Glycol 1,4-Cyclohexane Dimethylene Isosorbide Terephthalate) and Poly (1,4-Cyclohexane Dimethylene Isosorbide Terephthalate)

Duy-Nam Phan [1], Hoik Lee [1,*], Dongeun Choi [1], Chang-Yong Kang [2], Seung Soon Im [3] and Ick Soo Kim [1,*]

[1] Nano Fusion Technology Research Group, Division of Frontier Fibers, Institute for Fiber Engineering (IFES), Interdisciplinary Cluster for Cutting Edge Research (ICCER), Shinshu University, Ueda, Nagano 386-8567, Japan; duynamphan@gmail.com (D.-N.P.); g0024004@yahoo.co.jp (D.C.)

[2] Department of Metallurgical Engineering, Pukyong National University, Busan 608-739, Korea; metkcy@pknu.ac.kr

[3] Department of Organic and Nano Engineering, College of Engineering, Hanyang University, Seoul 133-791, Korea; imss007@hanyang.ac.kr

* Correspondence: hoik0822@gmail.com (H.L.); kim@shinshu-u.ac.jp (I.S.K.); Tel.: +81-80-9571-5148 (H.L.); +81-80-3088-0295 (I.S.K.)

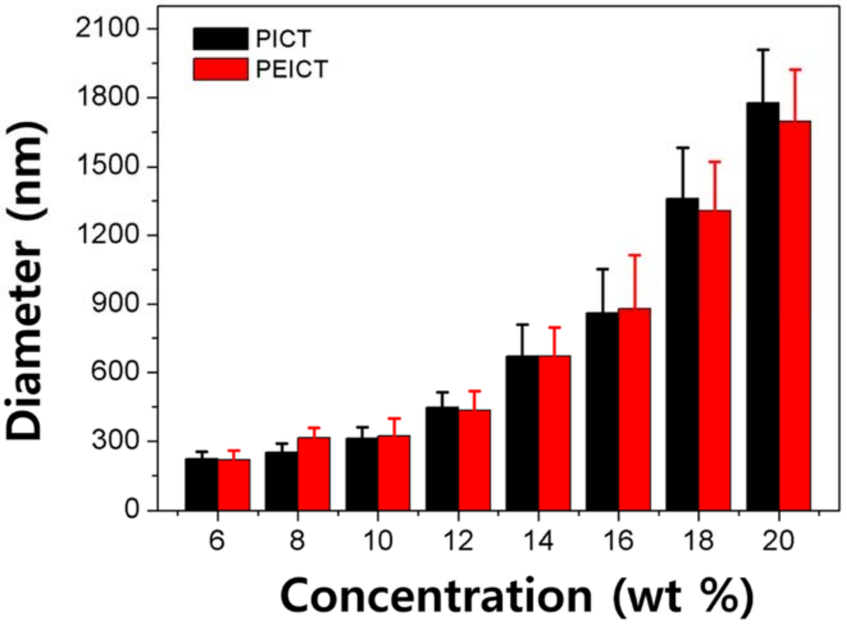

Figure S1. Correlation of nanofiber average diameter with concentration of PEICT and PICT solutions.

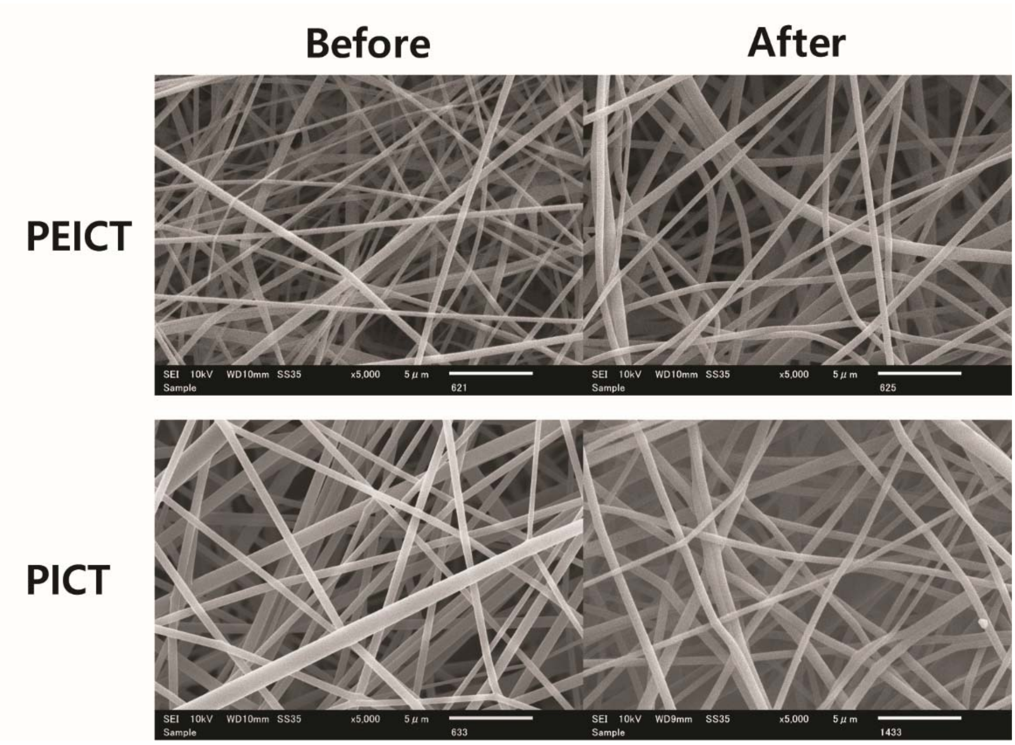

Figure S2. Morphology of PEICT and PICT nanofibers before and after annealing at 180 °C for 3 hrs.
